# READD‐ADSP Kenya: Opportunities for recruitment of participants for Alzheimer’s Disease genetic research in Sub‐Saharan Africa

**DOI:** 10.1002/alz.086706

**Published:** 2025-01-09

**Authors:** Christine W. Musyimi, Victoria Mutiso, Rufus O. Akinyemi, Raj Kalaria, David Ndetei

**Affiliations:** ^1^ Africa Mental Health Research and Training Foundation, Nairobi Kenya; ^2^ College of Medicine, University of Ibadan, Ibadan, Oyo State Nigeria; ^3^ Neuroscience and Aging Research Unit, Institute of Advanced Medical Research and Training, College of Medicine, University of Ibadan, Ibadan Nigeria; ^4^ Newcastle University, Newcastle upon Tyne United Kingdom; ^5^ University of Nairobi, Nairobi Kenya; ^6^ University of Nairobi, Nairobi, Nairobi Kenya; ^7^ Africa Mental Health Research and Training Foundation, Nairobi, Nairobi Kenya

## Abstract

**Background:**

The recruitment of individuals for Alzheimer’s disease (AD) genetic studies particularly those with low socioeconomic status, and living in rural areas remains a challenge in Sub‐Saharan Africa (SSA), due to stigma‐related cultural beliefs that hinder their participation. The Recruitment and Retention of Alzheimer’s Disease Diversity Genetic Cohorts in the ADSP (READD ‐ ADSP) project is a case‐control genetic epidemiological study involving individuals who are living with AD and disease ‐ free healthy control individuals. The aim is to build a resource that greatly expands Alzheimer’s disease genetic studies in the currently underrepresented African ancestry populations and Hispanic/Latinx individuals.

**Method:**

The READD‐ADSP study uses a comprehensive and community‐sensitive approach towards recruitment. We employed a four‐level systematic community approach to recruitment of cases of AD and matching healthy controls of individuals aged 60 years and above. We employed the following steps: (i) Creating awareness and reducing stigma through implementation of a dementia anti‐stigma intervention; (ii) Dementia screening to promote early detection of dementia, (iii) Clinician confirmatory diagnosis, and (iv) Ascertainment of AD through a battery of neuropsychological assessments by a clinician and psychologist.

**Result:**

Stigma reduction efforts resulted in reduction in negative beliefs and attitudes related to treatment, living well with dementia and care for the general public. This provided an opportunity for Community health workers to screen 3,546 older adults for dementia in community settings in Makueni County, Kenya. Confirmatory diagnosis was provided by a mental health nurse, resulting in a database for recruitment of 200 cases of AD and 200 controls to understand AD genetic architecture. By leveraging on this community‐based participatory research approach, 23 cases of AD and 21 controls have been recruited within the READD‐ADSP project in Kenya.

**Conclusion:**

The adoption and implementation of novel and systematic recruitment strategies in SSA could address local barriers to recruitment, improve best practices in AD research and ultimately advance development of targeted interventions in underrepresented community settings. This context‐specific approach could also allow researchers to be more fully empowered to respond to challenges within the recruitment process in order to enhance enrolment and retention low‐resource settings.

